# Serum and Glucocorticoid-Inducible Kinase 3/Nedd4-2 Signaling Pathway Participates in Podocyte Injury by Regulating the Stability of Nephrin

**DOI:** 10.3389/fphys.2021.810473

**Published:** 2022-01-20

**Authors:** Qing-Qing Dong, Zi-Fang Li, Hui Zhang, Hua-Pan Shu, Yu-Chi Tu, Qian-Qian Liao, Li-Jun Yao

**Affiliations:** ^1^Department of Nephrology, Union Hospital, Tongji Medical College, Huazhong University of Science and Technology, Wuhan, China; ^2^Blood Purification Center, Hubei No. 3 People’ Hospital of Jianghan University, Wuhan, China

**Keywords:** SGK3, nephrin, ubiquitin-proteasome degradation, Nedd4-2, adriamycin, podocytes

## Abstract

Serum and glucocorticoid-inducible kinase 3 (SGK3) is involved in maintaining podocyte function by regulating the protein levels of podocin and CD2-associated protein. Nephrin is also one of the slit diaphragm proteins of podocytes, but whether SGK3 participates in podocyte injury by regulating the levels of nephrin remains unclear. In this study, we focused on whether SGK3 affects nephrin levels and the mechanisms involved in the same. In the kidneys of adriamycin (ADR)-induced podocyte injury mouse model, the protein levels of SGK3 and nephrin were significantly decreased. Furthermore, the expression of SGK3 was negatively correlated with the output of proteinuria, and positively correlated with the levels of nephrin. In ADR-treated conditionally immortalized mouse podocyte cells (MPCs), the protein levels of nephrin and SGK3 were inhibited, while the constitutive expression of SGK3 reversed the ADR-induced decline in nephrin protein levels. Furthermore, ADR treatment or SGK3 inactivation enhanced the ubiquitin-proteasome degradation of nephrin in MPCs, and dramatically activated downstream effector proteins of SGK3, neural precursor cells expressing developmentally downregulated protein 4 subtype 2 (Nedd4-2) and glycogen synthase kinase-3 β (GSK3β). Similarly, Nedd4-2 or GSK3β overexpression resulted in increased activity of Nedd4-2 or GSK3β, and significantly downregulated nephrin levels. Interestingly, ubiquitin-mediated protein degradation of nephrin was regulated by Nedd4-2, rather than by GSK3β. In summary, SGK3 inactivation downregulated the levels of nephrin by increasing Nedd4-2 and GSK3β activity in ADR-induced podocyte injury model; in particular, the SGK3/Nedd4-2 signaling pathway was found to be involved in ubiquitin-mediated proteasome degradation of nephrin.

## Introduction

The kidney filtration barrier plays an important role in preventing the passage of macromolecules into the urinary space. Podocytes are the last layer of the filtration barrier and have numerous membrane processes. The adjacent foot processes of podocytes forms a slit diaphragm (Scott and Quaggin, 2015; New et al., 2016). Nephrin is the first identified slit diaphragm protein that forms a filter pore with selective permeability through the *cis* and *trans* interactions of its extracellular domains (Tryggvason et al., 2006; Martin and Jones, 2018). Mutations in the *NPHS1* gene that encodes nephrin, are characterized by congenital nephrotic syndrome and inactivation of nephrin, which results in the disappearance of the slit diaphragm, large amounts of proteinuria, and death in mice (Kestila et al., 1998; Ruotsalainen et al., 1999). While it has been reported that nephrin can be endocytosed *via* a raft-mediated endocytic pathway and the SUMOylation of nephrin also regulates its distribution and transformation on the cell membrane (Qin et al., 2009; Quack et al., 2011; Babayeva et al., 2013; Tossidou et al., 2014), the molecular mechanism of nephrin degradation has not been studied in detail.

Serum and glucocorticoid-inducible kinase 3 (SGK3) is a serine/threonine protein kinase involved in the regulation of various cellular functions such as cell proliferation, gene transcription, and ion transport (McCormick et al., 2004; Lang et al., 2006; He et al., 2011). Our previous study reported that the activity of SGK3 was decreased in podocyte injury models both *in vitro* and *in vivo*, and SGK3 knockout mice presented with proteinuria (Peng et al., 2018). Moreover, our studies revealed that SGK3 is involved in podocyte injury by inhibiting the expression of podocin and CD2-associated protein (CD2AP) (Peng et al., 2018). Given that nephrin forms a complex with podocin and CD2AP and podocin is also involved in nephrin signaling conduction (Boute et al., 2000; Schwarz et al., 2001; Huber et al., 2003; Lehtonen et al., 2005), it is unknown whether SGK3 participates in podocyte injury by regulating nephrin.

SGK3 can inactivate neural precursor cells expressing developmentally downregulated protein 4 subtype 2 (Nedd4-2) and glycogen synthase kinase-3β (GSK3β) *via* phosphorylation (Dai et al., 2002; Yuan et al., 2018). Nedd4-2 is a member of the E3 ubiquitin ligase that mediates substrate protein ubiquitination mediated degradation by transferring ubiquitin from its homologous to the E6-AP carboxyl terminus (HECT) domain to substrate proteins (Abriel and Staub, 2005; Rotin and Kumar, 2009). GSK3β participates in podocyte damage by affecting podocyte actin skeleton destruction and short foot mutations (Xu et al., 2014; Guo et al., 2016; Li et al., 2016). Previous reports have demonstrated that GSK3β regulates the nuclear transcription of nephrin through the transcriptional repressor *Snail* (Matsui et al., 2007; Wan et al., 2016; Wang et al., 2017). Our previous study showed that the SGK3/Nedd4-2 signaling pathway was involved in maintaining the stability of the podocalyxin/ezrin complex by affecting ezrin ubiquitination in podocytes (Yuan et al., 2018). However, it remains to be determined whether SGK3/GSK3β or SGK3/Nedd4-2 signaling pathway regulates nephrin protein levels.

Therefore, in this study, we investigated the effect of SGK3/Nedd4-2 and SGK3/GSK3β signaling pathways on the levels and ubiquitination of nephrin in podocyte injury induced by adriamycin (ADR) stimulation.

## Materials and Methods

### Reagents and Antibodies

Polyclonal rabbit anti-SGK3, anti-phospho-Nedd4-2 (Ser342), anti-Nedd4-2 and anti-phospho-GSK3(α/β) antibodies for immunoblotting and immunofluorescence were purchased from Cell Signaling Technology, Inc., (Beverly, MA, United States). Polyclonal rabbit anti-SGK3 antibody for immunohistochemistry was purchased from Abnova (Taipei, Taiwan, China). Monoclonal mouse anti–phospho-GSK3(α/β) antibody for immunoblotting, Polyclonal rabbit anti-SGK3 antibody for immunofluorescence, and monoclonal mouse anti-nephrin antibody for ubiquitination and immunofluorescence were purchased from Santa Cruz Biotechnology (Santa Cruz, CA, United States). Polyclonal rabbit nephrin for antibody immunoblotting and immunohistochemistry was purchased from boster (Wuhan, China). Monoclonal mouse anti-ubiquitin (UB) antibody was purchased from Covance Inc., (UT, United States). Monoclonal mouse anti-GAPDH, rabbit polyclonal anti-Na,K-ATPase, rabbit polyclonal anti-flag tag, horseradish peroxides-conjugated anti-rabbit and anti-mouse secondary antibodies for immunoblotting, Cy3–conjugated Affinipure Goat Anti-Mouse IgG (H + L) and Fluorescein (FITC)–conjugated Affinipure Goat Anti-Rabbit IgG (H + L) were purchased from Proteintech (Wuhan, China). 4,6-diamidino-2-phenylindole (DAPI) was from Beyotime. The pcDNA3.1/mSGK3-S486D plasmid, pcDNA3/mNedd4-2 plasmid and pMO/mSGK3-K191M plasmid were gifts from David Pearce (Departments of Medicine, and Molecular and Cellular Pharmacology, University of California, San Francisco, CA, United States). The pCMV/HA/mGSK3β plasmid was purchased from Origene Technology, Inc., (Rockville, MD, United States). The ubiquitin-Flag (UB-Flag) plasmid was a gift from Prof. Duan Qiuhong, Biochemistry Laboratory, Tongji Medical College, Huazhong University of Science and Technology. Recombinant lentivirus *SGK3*-shRNA and *Nedd4-2*-shRNA were purchased from Genechem (Shanghai, China). MG132 were purchased from APExBIO Technology (Houston, TX, United States). Phosphatase inhibitor mixture and proteinase inhibitor cocktail were purchased from Roche Applied Science (Indianapolis, IN, United States).

### Adriamycin Nephritis Mouse Model

Male BALB/c mice were purchased from the Animal Experiment Center of Wuhan University (Wuhan, China). Baseline urine was collected in the male BALB/C mice (20–25 g, 8 weeks old). 8-week-old mice were treated with a single-dose tail vein injection of 10.5 mg/kg ADR (Sigma, USA, D1515) after 1 week of adaptive breeding (ADR group, *n* = 6). Control mice were treated with an equivalent volume of phosphate-buffered saline (PBS) (control group, *n* = 5). All mice were euthanized at 4 weeks after ADR treatment, and blood samples and kidneys were collected. The harvested kidneys were collected for western blotting, immunohistochemical and immunofluorescence analysis. All the animal experiments were approved by the Animal Care and Use Committee of Tongji Medical College. All methods were performed in accordance with the relevant guidelines and regulations.

### Measurement of Urine and Serum Biochemistry

Blood urea nitrogen (BUN) and urinary and serum creatinine levels were determined by the respective kits (Nanjing Jiancheng Bioengineering Institute, Nanjing, China), and total protein levels in urine were assessed by Urinary Total Protein Determination Kit (Ningbo Elejech Biologic Technology, Zhejiang, China), according to the manufacturer’s instruction. The urinary albumin excretion rate was expressed as the ratio of albumin to creatinine.

### Morphometric Analysis, Immunohistochemistry, and Immunofluorescence of the Mouse Kidney

Renal tissues were fixed in 10% formalin neutral fixative overnight after removal, embedded in paraffin and sliced to make 5-μm-thick sections that were stained with periodic acid-Schiff (PAS) reagent for histopathology. Immunohistochemistry and immunofluorescence staining proceeded and analyzed essentially as previously described (Peng et al., 2018). The sections was subject to rehydration by soaking in xylene for 30 min for two intervals and then placed into progressively lower concentrations of ETOH (100, 95, 70, and 50%) for 5 min each and finally placed in ddH20 for 5 min. Antigen retrieval was performed by incubating slides in a citrate buffer solution for 2 min in a 95°C water bath. Slides were cooled at room temperature for 20 min and washed 3 times with PBS. Endogenous peroxidase activity was blocked with 3% H2O2 in methanol for 30 min. Non-specific binding was blocked using 10% normal goat serum for 30 min. The slides were washed with PBS and incubated with polyclonal anti-SGK3, polyclonal anti-nephrin and anti-Nedd4-2 overnight at 4°C.

For immunohistochemistry, the sections were incubated with polyperoxidase anti-rabbit and anti-goat IgG secondary antibodies for 30 min at room temperature The bound antibody was visualized using a DAB Kit. Positive expression for SGK3 and nephrin in 50 random glomeruli per mouse were counted and expressed as average per glomerulus. All images were acquired using light microscopy. Positive expression for SGK3 and nephrin were semi-quantitative using the Image Pro Plus software.

For immunofluorescence, the sections were stained with Cy3–conjugated Affinipure Goat Anti-Mouse IgG (H + L) (SA00009-1; Proteintech, Wuhan, China) and Fluorescein (FITC)–conjugated Affinipure Goat Anti-Rabbit IgG (H + L) (SA00003-2, Proteintech, Wuhan, China). Nuclei were co-stained with 4,6-diamidino-2-phenylindole (DAPI) for 5 min. All sections were observed under a fluorescent microscope (Nikon, Tokyo, Japan).

### Cell Culture and Chemical Treatment

The conditional immortalized mouse podocytes were cultivated as described earlier (Peng et al., 2018). Briefly, cells were provided with RPMI 1640 medium (HyClone, United States) supplemented with 10% (v/v) fetal bovine serum (GIBCO, Thermo Fisher Scientific Inc., United States), 100 μg/ml streptomycin and 100 U/ml penicillin. Cells were cultured at 33°C under 5% CO2 and 95% air in culture medium supplemented with 10 U/ml recombinant interferon-γ (IFN-γ) in order to proliferation. Podocytes were transferred at 37°C in IFN-γ-free medium cultivated 10–14 days for inducing differentiation. Mouse podocytes cells were treated with ADR (0.2 μg/ml) for 24 h or MG132 (10 μM) for 8 h at 37°C before collected.

### Plasmid Transfection and Recombinant Lentivirus Infection

Mouse podocytes were transfected with various plasmids for 48 h using Lipofectamine 2000 reagent in line with the manufacturer’s instructions (Invitrogen, Carlsbad, CA, United States), and the cell lysates were prepared for immunoblot and ubiquitination experiment measurements. Mouse podocytes were infected with *SGK3* and *Nedd4-2* short hairpin RNAs (shRNAs) or scramble lentiviruses for seventy 2 h and then cell lysates were collected for immunoblot.

### Membrane Protein Extraction and Western Blot Analysis

Protein expression was measured by western blot analysis. Mouse podocytes were lysed in Lysis buffer added with protease inhibitors cocktail and dithiotreitol (DTT). Membranous protein was extracted according to the manufacturer’s instructions (KeyGEN BioTECH, Jiangsu, China). In brief, the suspension kept shaking for 30 s 6 times after every 1 min, and then discarded after centrifugation. The precipitate was added with extraction buffer and kept shaking for 30 s six times after every 5 min. The supernatant protein was membrane protein. For total protein, the mouse podocytes and kidney tissues were lysed in RIPA buffer supplemented with a protease inhibitor cocktail, PMSF and phosphatase inhibitor for 1 h, and the supernatant was extracted as total protein. Equal amounts of proteins were separated by electrophoresis on SDS-polyacrylamide gel electrophoresis (SDS-PAGE) gels then transferred to a polyvinylidene fluoride membrane (Millipore, Bedford, MA, United States). Membranes were blocked with 5% milk for 2 h at room temperature and incubated with appropriate primary antibody at 4°C overnight. The following day membranes were incubated with corresponding horseradish peroxidase-conjugated secondary antibodies for 1 h at room temperature and detected by ECL (Millipore, Germany).

### Ubiquitination Assay

Ubiquitination assays were performed as described (Yuan et al., 2018). Mouse podocytes were treated with ADR (0.2 μg/ml) or transfected with plasmids and MG132 (10 μM) was added for 8 h before collected to prevent proteasomal degradation. Cells were washed with PBS three times and dissociated in 1 ml lysis buffer [50 mM HEPES (pH 7.5), 150 mM NaCl, 1 mM EGTA, 1% TritonX-100, 25 mM sodium fluoride, 40 mM β-glycerolphosphate, 10 mM sodium pyrophosphate, 1.5 mM MgCl2, 10% glycerol] supplemented with PMSF, cocktail, and phosphatase inhibitors. The lysates were centrifuged at 12,000 rpm for 15 min at 4°C. The supernatant proteins (about 1 mg) were formulated into an equal volume system and added to the protein A/G agarose beads for co-immunoprecipitation using the appropriate primary antibody (anti-Flag antibody, anti-nephrin antibody) overnight at 4°C. Agarose beads were gathered and loading buffers (5×) were added to the co-immunoprecipitates. The samples were separated by SDS-polyacrylamide gels for western blot and immunoblotted with anti-nephrin antibody or anti-ubiquitin (UB) antibody.

### Co-immunoprecipitation Assays

Mouse podocytes were grown on 10-cm dishes, and transfected with 10 μg of the plasmids DNA encoding mouse Nedd4-2 or empty vector using Lipofectamine 2000 reagent for 48 h. After transfection, cells were washed twice at 4 °C in cold PBS and dissociated in 500 μl IP lysis buffer (Beyotime Biotechnology, Shanghai, China). After centrifugation (12,000 rpm, 4°C, 15 min), the supernatant was quantified by Bradford assay and 1 mg of lysate was incubated with the anti-nephrin antibody and protein A/G agarose beads. Then the samples were subjected to western blot analysis.

### Query for Substrate-NPHS1 (Nephrin) Interactions in UbiBrowser

UbiBrowser^1^ is an integrated bioinformatics platform, which is used to predict proteome-wide human E3-substrate networks. Totally 20,220 human proteins’ corresponding E3 ligases can be explored. Furthermore, 4,560 human proteins’ 19,451 ubiquitination sites can also be explored (Li et al., 2017). We used it to predict the potential E3 ligases of NPHS1 (nephrin).

### Statistical Analysis

All data were expressed as the mean ± SE and SPSS 22.0 software was used for analyzed. An unpaired Student’s *t* test was used for comparison of two groups and a one-way ANOVA with Dunnett’s *post hoc* test was applied for comparison of more than 2 groups. The correlation of SGK3 expression with proteinuria or with nephrin expression was analyzed by Spearman’s rank correlation coefficient. A value of *P* < 0.05 was defined statistically significant. Analyses were performed using Graphpad Instat software (GraphPad Software, San Diego, CA, United States).

## Results

### SGK3 Expression Is Downregulated and Negatively Correlated With Proteinuria in the Adriamycin Nephritis Mouse Model

**FIGURE 1:**
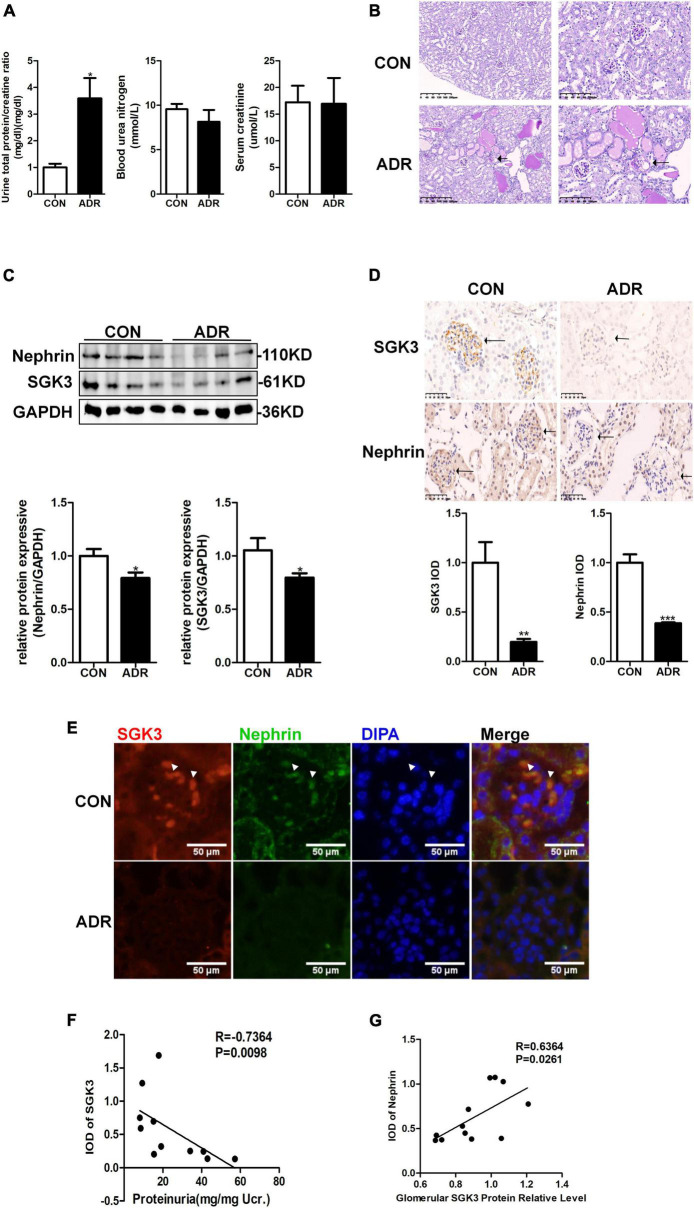
SGK3 was associated with proteinuria and nephrin expression in ADR nephritis. An intravenous injection of ADR (10.5 mg/kg) was used to induce nephritis mouse model. **(A)** BUN, serum creatinine, and the urinary albumin/creatinine ratio were measured in mice after treatment with ADR 28 days. **(B)** Morphologically, PAS staining show tubules were frequently dilated and contained some casts after ADR injection. **(C)** Top panel: immunoblot analysis for nephrin and SGK3, GAPDH was used for normalization. Bottom panel: Quantification of nephrin and SGK3 by densitometry. **(D)** Top panel: immunohistochemical staining shows SGK3 and nephrin expression in the glomeruli after ADR injury. Representative images from six mice in each group are shown. Bottom panel: computerized morphometric quantification of SGK3 and nephrin staining in the glomeruli of control and ADR mice. **(E)** Representative immunofluorescent images showed staining with specific antibodies to SGK3 (red) and nephrin (green) in the glomeruli of control and ADR mice. Arrowheads indicate the colocalization of SGK3 and nephrin. **(F)** There was a significantly negative correlation between SGK3 expression and proteinuria levels in BALB/c mice (*n* = 5 in control group, *n* = 6 in ADR group). **(G)** There was a significantly positive correlation between SGK3 and nephrin expression in BALB/c mice (*n* = 5 in control group, *n* = 7 in ADR group). *P < 0.05, **P < 0.01, ***P < 0.001 vs. control group.

Our previous study demonstrated that the absence of SGK3 expression leads to podocyte dysfunction and proteinuria (Peng et al., 2018). To determine the relationship between SGK3 and proteinuria in ADR nephritis, an ADR nephritis mouse model was established as previously described (Peng et al., 2018). As shown in Figure 1A, on the 28th day after modeling, urinary protein excretion was significantly increased in the ADR mice compared with that in the control group. However, no significant difference in blood urea nitrogen and serum creatinine levels was noted. Furthermore, the renal tissue presented obvious pathological damage by periodic acid-Schiff staining after ADR injection. Protein casts were found in the renal tubules and glomerular focal segmental sclerosis was visible (Figure 1B). Western blotting analysis revealed a significant decrease in the protein level of nephrin and SGK3 in the kidneys of ADR nephritis mice (Figure 1C). Immunohistochemical analysis of kidney tissue sections revealed that SGK3 and nephrin levels were significantly lower in the glomeruli of ADR mice than those of the control mice (Figure 1D). Immunofluorescence examination presented that SGK3 was expressed in the podocytes of mouse kidney, and ADR treatment decreased the co-localization of SGK3 and nephrin (Figure 1E). Correlation analysis showed that the levels of SGK3 were negatively correlated with the urinary output of the protein (Figure 1F). Furthermore, there was a significant positive correlation between the levels of SGK3 and nephrin (Figure 1G). Thus, our results showed that SGK3 participated in ADR-induced podocyte injury, possibly by affecting nephrin levels.

### SGK3 Is Involved in Adriamycin-Induced Downregulation of Nephrin *in vitro*

**FIGURE 2:**
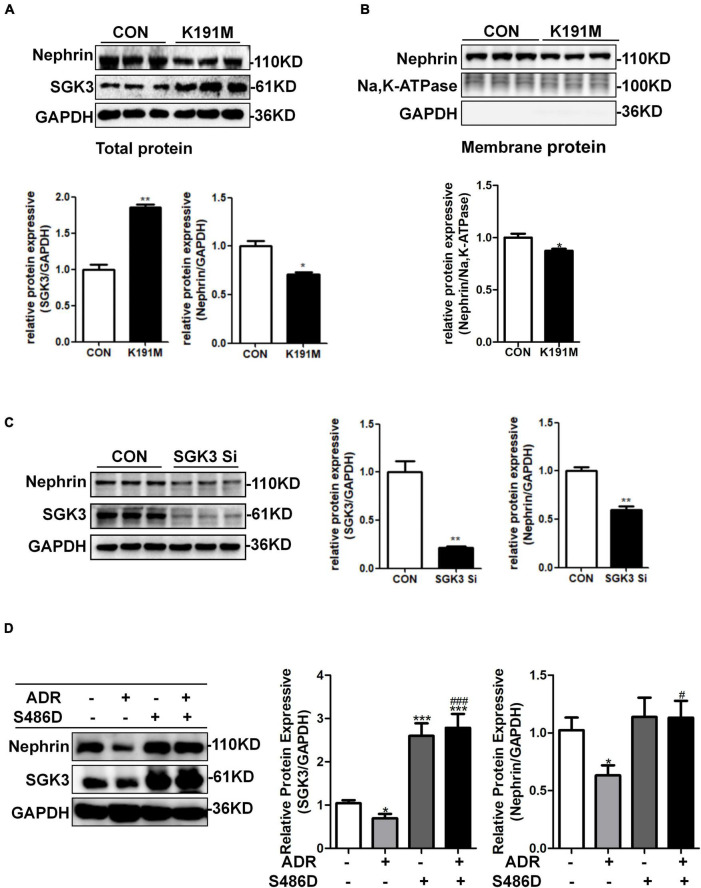
SGK3 was involved in ADR-induced downregulation of nephrin. Mouse podocytes were transiently transfected with SGK3-K191M plasmid for 48 h. **(A)** Top panel: immunoblot analysis for nephrin and SGK3 expression in total protein, GAPDH was used for normalization. Bottom panel: Quantification of nephrin and SGK3 by densitometry. **(B)** Top panel: immunoblot analysis for nephrin in membrane protein, Na,K-ATPase was used for normalization. **(C)** MPCs were transiently infected with *SGK3*-shRNA for 72 h. Left panel: immunoblot analysis for nephrin and SGK3 expression; GAPDH was used for normalization. Right panel: Quantification of nephrin and SGK3 by densitometry.*P < 0.05, **P < 0.01 vs. control group. **(D)** MPCs were transiently transfected with SGK3-S486D plasmid for 48 h and ADR was applied at last 24 h. Left: immunoblot analysis for nephrin and SGK3 expression; GAPDH was used for normalization. Right: Quantification of nephrin and SGK3 by densitometry. *P < 0.05, **P < 0.01, ***P < 0.001 vs. ADR-/S486D- group, ^#^*P* < 0.05, ^###^*P* < 0.001 vs. ADR + /S486D- group.

We have previously reported that SGK3 is involved in the downregulation of podocin and CD2AP expression and podocyte viability is induced by puromycin aminonucleoside (PAN) (Peng et al., 2018). However, it remains unclear whether SGK3 participates in an ADR-mediated decrease in nephrin levels. To address this question, podocytes were transfected with an SGK3 inactivated plasmid, SGK3-K191M, or *SGK3*-specific shRNAs *in vitro*. We found that the total and membrane protein levels of nephrin were significantly downregulated after SGK3 inactivation (Figures 2A,B) and knockdown (Figure 2C), indicating that downregulation of SGK3 led to decreased nephrin protein levels. We also transfected mouse podocyte cells (MPCs) with an SGK3 constitutively activated plasmid, SGK3-S486D, followed by ADR treatment. We noted that the increase in SGK3 activity reversed the ADR-induced decrease in nephrin levels (Figure 2D), implying that SGK3 was involved in the ADR-mediated decrease in nephrin levels.

### Adriamycin Increases Ubiquitin-Mediated Protein Degradation of Nephrin

**FIGURE 3:**
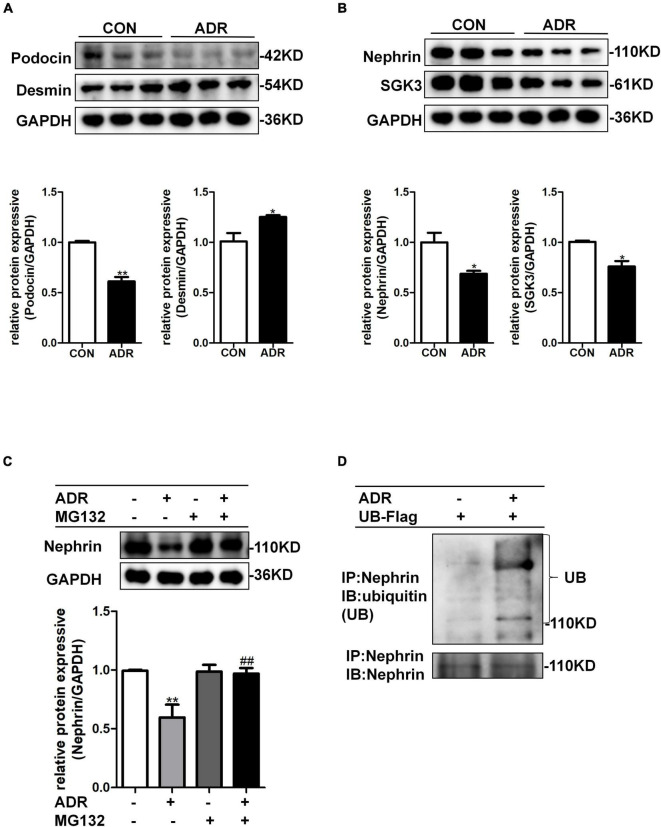
ADR treatment leaded to increased ubiquitination degradation of nephrin. Mouse podocytes were treated with 0.2 μg/ml ADR for 24 h. **(A)** Top panel: immunoblot analysis for desmin and podocin, GAPDH was used for normalization. Bottom panel: Quantification of desmin and podocin by densitometry. **(B)** Top panel: immunoblot analysis for nephrin and SGK3, GAPDH was used for normalization. Bottom panel: Quantification of nephrin and SGK3 by densitometry. Data are means ± SE of three independent experiments. *P < 0.05, **P < 0.01 vs. control group. **(C)** MPCs were treated with ADR (0.2 μg/ml) and then treated with or without MG132 for 8 h. Top panel: immunoblot analysis for nephrin expression. Bottom panel: Quantification of nephrin by densitometry. **P < 0.01 vs. ADR-/MG132- group, ^##^*P* < 0.01 vs. ADR + /MG132- group. **(D)** MPCs were transiently transfected with ubiquitin-Flag (UB-Flag) plasmid for 48 h then ADR was added for 24 h and MG132 was applied at last 8 h. Whole-cell lysates were precipitated with an anti-nephrin antibody and the immunoprecipitates were blotted with anti-ubiquitin and anti-nephrin antibodies.

Multiple studies have shown that ADR stimulation leads to podocyte injury (Liu et al., 2018; Angeletti et al., 2020). As illustrated by western blotting, ADR treatment indeed resulted in increased levels of podocyte damage marker, desmin, and decreased levels of podocin (Figure 3A), thereby validating the podocyte damage model. As shown in Figure 3B, the levels of nephrin and SGK3 decreased after ADR treatment. We further explored the effect of ADR on nephrin degradation. The cultured MPCs were treated with ADR and the proteasome inhibitor, MG132. Compared with the ADR + /MG132- group, the levels of nephrin in the ADR + /MG132 + group were significantly increased to the control level. Thus, ADR-induced downregulation of nephrin protein levels could be reversed by MG132 (Figure 3C). Next, we determined the ubiquitination level of nephrin after ADR treatment. MPCs were transfected with a ubiquitin-Flag (UB-Flag) plasmid for 48 h and treated with ADR for 24 h. As expected, the ubiquitin-mediated protein degradation of nephrin was enhanced in the ADR-treated MPCs (Figure 3D). These data suggested that ADR treatment increased the ubiquitination mediated degradation of nephrin.

### SGK3 Triggers Ubiquitin-Mediated Degradation of Nephrin in Cultured Mouse Podocyte Cells

**FIGURE 4:**
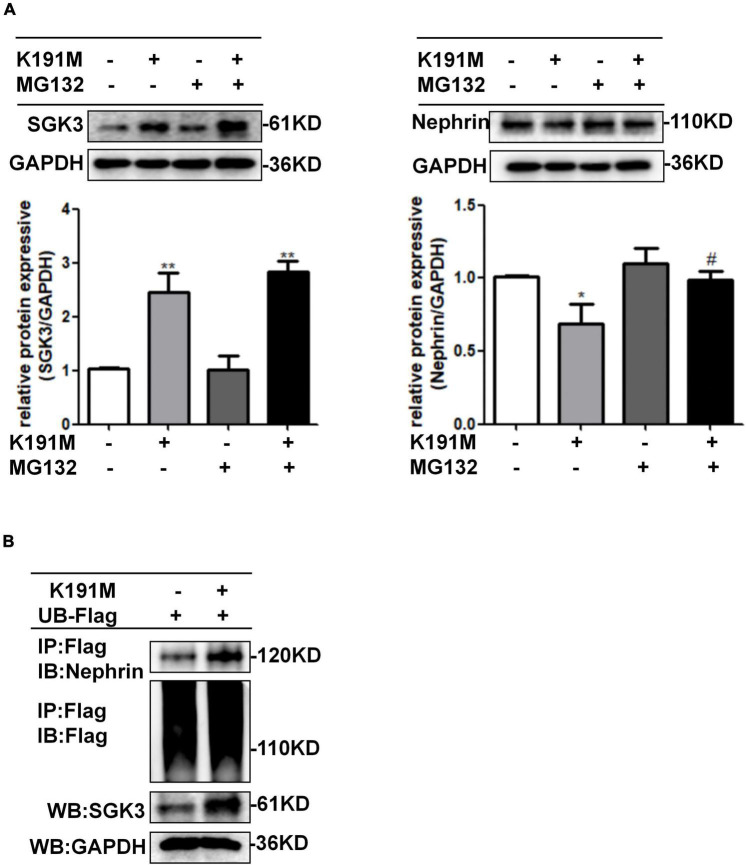
SGK3 inactivation leaded to increase ubiquitination degradation of nephrin. **(A)** MPCs were transiently transfected with SGK3-K191M plasmid for 48 h and MG132 was applied at last 8 h. Left: immunoblot analysis and quantification for SGK3 expression; GAPDH was used for normalization. Right: immunoblot analysis and quantification for nephrin expression; GAPDH was used for normalization. *P < 0.05, **P < 0.01 vs. K191M-/MG132- group, ^#^*P* < 0.05 vs. K191M + /MG132- group. **(B)** MPCs were transiently co-transfected with SGK3-K191M and ubiquitin-Flag (UB-Flag) plasmids for 48 h and MG132 was applied at last 8 h. Whole-cell lysates were precipitated with an anti-Flag antibody and the immunoprecipitates were immunoblotted with anti-nephrin and anti-Flag antibodies. The protein expression levels of SGK3 were measured in the WCLs with immunoblot assays.

To further investigate the mechanism by which SGK3 regulates nephrin protein levels, we transfected podocytes with SGK3-K191M and then treated the cells with or without MG132. The decreased nephrin levels in the K191M + /MG132- group were reversed in the K191M + /MG132 + group (Figure 4A), suggesting that SGK3 inactivation decreased nephrin protein levels by promoting the proteasome degradation pathway of nephrin. Next, we examined the effect of SGK3 on nephrin ubiquitination. Cultured MPCs were co-transfected with SGK3-K191M and ubiquitin-Flag (UB-Flag) plasmids, and the ubiquitination level of nephrin was assessed by a ubiquitination assay. As shown in Figure 4B, the ubiquitination level of nephrin was significantly increased in the SGK3-K191M transfection group compared with that in the control group. These studies suggested that the downregulation of SGK3 activity led to an increased ubiquitin-proteasome-mediated protein degradation of nephrin.

### SGK3/Nedd4-2 Signaling Pathway Modulates Nephrin Levels Through the Ubiquitin–Proteasome Degradation Pathway

**FIGURE 5:**
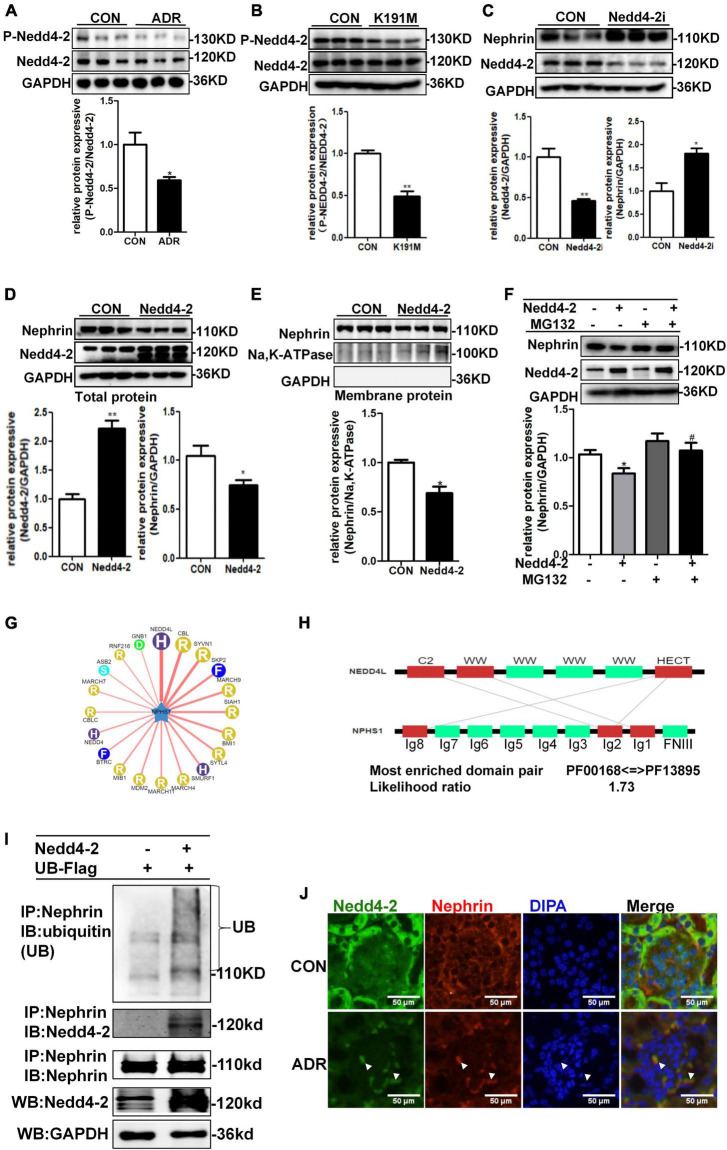
SGK3/Nedd4-2 signaling pathway regulated protein expression of nephrin through the ubiquitin-proteasome pathway. **(A)** MPCs were treated with 0.2 μg/ml ADR for 24 h. Top panel: immunoblot analysis for phospho-Nedd4-2 and Nedd4-2 expression. Bottom panel: Quantification of the ratio of phospho-Nedd4-2 to Nedd4-2 by densitometry. **(B)** MPCs were transiently transfected with SGK3-K191M plasmid for 48 h. Top panel: immunoblot analysis for phospho-Nedd4-2 and Nedd4-2 expression. Bottom panel: Quantification of the ratio of phospho-Nedd4-2 to Nedd4-2 by densitometry. **(C)** MPCs were infected with scramble (control) or *Nedd4-2*-specific shRNAs for 72 h. Top panel: immunoblot analysis for nephrin and Nedd4-2 expression; GAPDH was used for normalization. Bottom panel: Quantification of nephrin and Nedd4-2 by densitometry. **(D,E)** MPCs were transiently transfected with *Nedd4-2 WT* plasmid for 48 h. Top panel: immunoblot analysis for nephrin and Nedd4-2 expression; GAPDH was used for normalization. Bottom panel: Quantification of nephrin and Nedd4-2 by densitometry (D). Top panel: immunoblot analysis for nephrin in membrane protein, Na,K-ATPase was used for normalization (E). Bottom panel: Quantification of nephrin by densitometry. *P < 0.05, **P < 0.01 vs. control group. **(F)** MPCs were transiently transfected with *Nedd4-2 WT* plasmid for 48 h and MG132 was applied at last 8 h. Top panel: immunoblot analysis for nephrin and Nedd4-2 expression; GAPDH was used for normalization. Bottom panel: Quantification of nephrin and Nedd4-2 by densitometry. *P < 0.05 vs. Nedd4-2-/MG132- group, ^#^*P* < 0.05 vs. Nedd4-2 + /MG132- group. **(G)** Network view of predicted E3-substrate interactions in UbiBrowser web services. In network view, the central node is NPHS1 (nephrin), as a substrate, and the surrounding nodes are the predicted E3 ligases. The width of the edge reflects the confidence of the interaction. **(H)** The possible binding regions of NPHS1 (nephrin) and Nedd4-2. **(I)** Mouse podocytes were transiently transfected with *Nedd4-2 WT* plasmids for 48 h. Whole-cell lysates were precipitated with an anti-nephrin antibody and the immunoprecipitates were blotted with anti-Nedd4-2, anti-ubiquitin and anti-nephrin antibodies. The protein expression levels of Nedd4-2 in WCLs were analyzed with an immunoblot. **(J)** Representative immunofluorescent images showed staining with specific antibodies to Nedd4-2 (green) and nephrin (red) in the glomeruli of control and ADR mice. Arrowheads indicate the colocalization of Nedd4-2 and nephrin.

Previous report showed that Nedd4-2, a common E3 ubiquitin ligase, was abundantly expressed in the cytoplasm of podocytes of mouse kidney and contributed to dendrin degradation, which is also a component of the slit diaphragms (SDs) complex (Shirata et al., 2017). To explore the specific molecular mechanism underlying the regulation of nephrin by SGK3, we first examined whether the SGK3 target protein Nedd4-2 was involved in SGK3 triggered ubiquitin-mediated degradation of nephrin. We confirmed that the phosho-Nedd4-2/Nedd4-2 ratio was significantly downregulated after ADR treatment or SGK3-K191M plasmid transfection, which indicated that the activity of Nedd4-2 increased because phospho-Nedd4-2 was in an inactivated form (Figures 5A,B). This demonstrated the involvement of the SGK3/Nedd4-2 signaling pathway in the ADR-induced podocytes injury *in vitro*. We found that the protein levels of nephrin increased after Nedd4-2 knockdown (Figure 5C) and the total and membrane protein levels of nephrin decreased after Nedd4-2 overexpression (Figures 5D,E). Furthermore, MG132 administration could reverse the Nedd4-2 overexpression-induced decline in nephrin protein levels (Figure 5F), suggesting that Nedd4-2 was involved in the ubiquitin-mediated nephrin degradation. As there were no canonical WW-binding PPxY motifs, a classical target sequence of Nedd4-2, present in nephrin, we queried NPHS1 (nephrin) as a substrate in the web tool of UbiBrowser^2^ to explore the detailed mechanism of Nedd4-2 on nephrin degradation. We found that nephrin could interact with some of the E3 ligases, wherein Nedd4-2 showed the strongest interaction with nephrin (Figure 5G). Furthermore, Ig-like domains (Ig2 and Ig8) were predicted as the binding sites of NPHS1 (nephrin) to the HECT domain of Nedd4-2 (Figure 5H). Using a co-immunoprecipitation technique, we demonstrated that nephrin could indeed interact with Nedd4-2 (Figure 5I), and the ubiquitination levels of nephrin was significantly enhanced in the Nedd4-2-transfected mouse podocytes compared with that in the control mouse podocytes (Figure 5I). Immunofluorescence examination presented that Nedd4-2 was slightly expressed in the podocytes of mouse kidney, and ADR treatment resulted in increase co-localization of Nedd4-2 and nephrin (Figure 5J). These data suggested that the SGK3/Nedd4-2 signaling pathway was involved in ubiquitin-mediated protein degradation of nephrin.

### SGK3/GSK3β Signaling Pathway Regulates Nephrin Protein Expression

**FIGURE 6:**
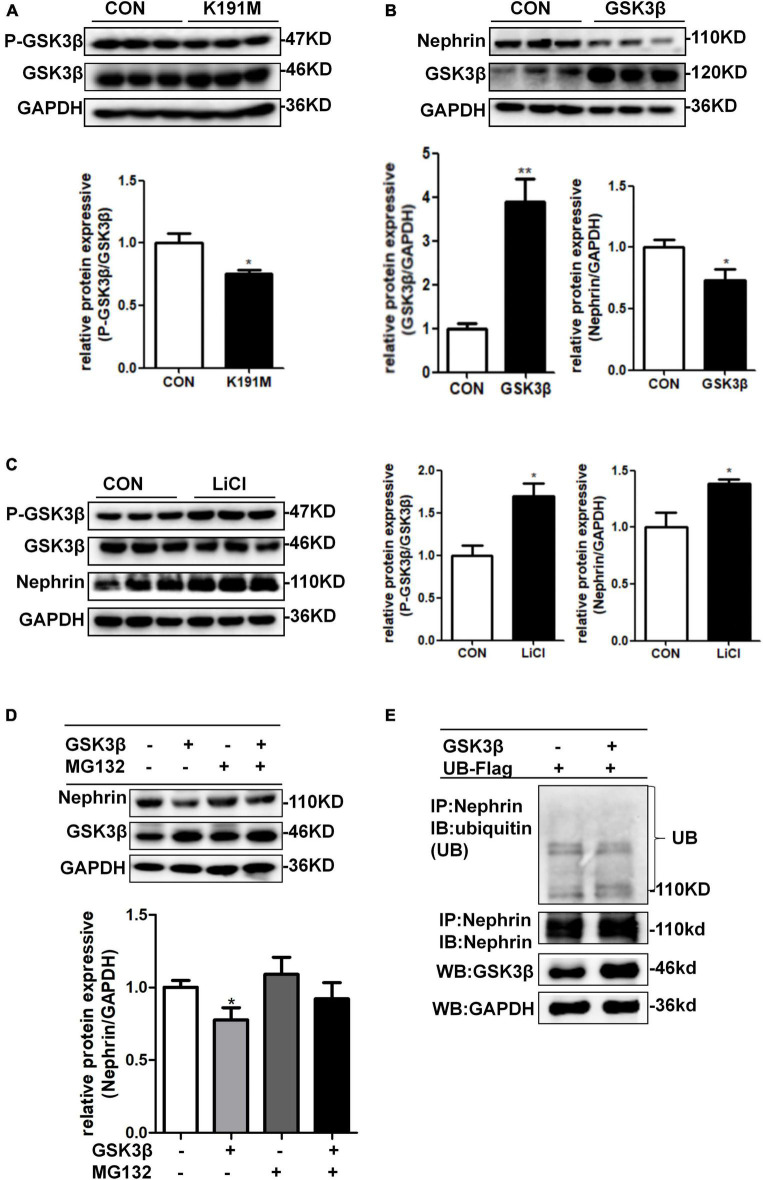
SGK3/GSK3β signaling pathway regulated protein expression of nephrin. **(A)** Mouse podocytes were transiently transfected with SGK3-K191M plasmid for 48 h. Top panel: immunoblot analysis for phospho-GSK3β and GSK3β expression. Bottom panel: Quantification of the ratio of phospho-GSK3β to GSK3β by densitometry. **(B)** MPCs were transiently transfected with *GSK3*β plasmid for 48 h. Top panel: immunoblot analysis for nephrin and GSK3β expression, GAPDH was used for normalization. Bottom panel: Quantification of nephrin and GSK3β by densitometry. **(C)** MPCs were treated with 10 μM LiCl for 24 h. Left panel: immunoblot analysis of phospho-GSK3β and GSK3β and nephrin expression. Right panel: Quantification of nephrin by densitometry. *P < 0.05, **P < 0.01 vs. control group. **(D)** MPCs were transiently transfected with *GSK3*β plasmid for 48 h and MG132 was applied at last 8 h. Top panel: immunoblot analysis for nephrin and GSK3β expression; GAPDH was used for normalization. Bottom panel: Quantification of nephrin and GSK3β by densitometry. *P < 0.05 vs. GSK3β-/MG132- group. **(E)** Mouse podocytes were transiently co-transfected with *GSK3*β and ubiquitin-Flag (UB-Flag) plasmids for 48 h and MG132 was applied at last 8 h. Whole-cell lysates were precipitated with an anti-nephrin antibody and the immunoprecipitates were immunoblotted with anti-ubiquitin and anti-nephrin antibodies. The protein expression levels of GSK3β in WCLs were analyzed by immunoblot.

We had previously reported that PAN stimulation and SGK3 shRNA infection of podocytes significantly decreased the phosphorylation of GSK3β, and the SGK3/GSK3β signaling pathway inhibited podocin expression during PAN-induced podocyte injury (Peng et al., 2018). In this study, we further explored whether the SGK3/GSK3β signaling pathway regulates nephrin levels. Consistent with our previous report, phosho-GSK3β/GSK3β ratio was significantly downregulated after SGK3-K191M plasmid transfection, which indicated that the activity of GSK3β increased because phospho-GSK3β was in an inactivated form (Figure 6A). Moreover, the levels of nephrin decreased in the GSK3β transfection group compared with that in the control group (Figure 6B), and the protein levels of nephrin were also significantly increased after treatment with LiCl, a GSK3β inhibitor (Peng et al., 2018), for 24 h (Figure 6C). In addition, when MG132 was added after *GSK3*β transfection, the protein levels of nephrin did not decrease (Figure 6D). However, nephrin ubiquitination levels were similar between the *GSK3*β-transfected mouse podocytes and the control mouse podocytes (Figure 6E). These results indicated that the SGK3/GSK3β signaling pathway regulated the protein levels of nephrin.

## Discussion

SGK3 is a serine/threonine protein kinase that is involved in regulating cell proliferation and ion transport (Lang et al., 2006; He et al., 2011). Our previous studies have shown that SGK3 inactivation inhibited podocyte cell viability, downregulated the expression of podocin and CD2AP proteins, and increased ubiquitination-mediated degradation of podocalyxin and ezrin (Peng et al., 2018; Yuan et al., 2018). The present study demonstrated that the decreased levels of SGK3 were positively correlated with the levels of nephrin in the kidneys of an ADR-nephritis mouse model. We also showed that SGK3 resulted in decreased levels of nephrin in an ADR-induced podocyte injury cell model. Moreover, both ADR treatment and SGK3 inactivation increased the ubiquitin-proteasome degradation of nephrin in cultured podocytes. In addition, SGK3 inactivation was also found to potentially regulate nephrin protein levels by increasing Nedd4-2 and GSK3β activity. In addition, SGK3 inactivation was also found to potentially regulate nephrin protein levels by increasing Nedd4-2 and GSK3β activity. In particular, SGK3/Nedd4-2, rather than the SGK3/GSK3β signaling pathway, participated in ADR-mediated nephrin ubiquitination. Thus, this study helps identify a new mechanism by which SGK3 potentially maintains the normal function of podocytes.

To the best of our knowledge, this is the first study to have shown that ADR stimulation enhances nephrin ubiquitination, a key component of slit diaphragm membranes (Oh et al., 2004; Qin et al., 2009; New et al., 2014, 2016). Previous reports have shown that ADR regulates nephrin protein levels through nuclear transcription (Yu et al., 2016; Huang et al., 2019; Wang et al., 2020). Meyer-Schwesinger et al. (2009) observed that the levels of ubiquitin-modifying enzyme ubiquitin C-terminal hydrolase-L1 in podocytes was increased in the biopsies of patients with membranous nephropathy, focal segmental glomerulosclerosis, and lupus nephritis IV. Tossidou et al. (2010) pointed out that the increased ubiquitination levels of podocytes could promote the internalization of slit diaphragm proteins nephrin, resulting in podocyte dedifferentiation, foot process fusion, and proteinuria. In this study, we demonstrated that the increased ubiquitination level of nephrin may be directly involved in ADR-induced MPC injury.

We also revealed that SGK3 inactivation triggered ADR-induced nephrin ubiquitination. The glomerular filtration barrier is highly sized and charge-selective. Previously, we have shown that SGK3 modulates the degradation of podocalyxin, a heavily sialylated membrane protein located on the apical surface of podocytes, resulting in damage to the glomerular charge barrier (Takeda et al., 2000). In the present study, SGK3 was found to participate in the degradation of the slit diaphragm protein nephrin, that contributes significantly to the preservation of the molecular filtration barrier (Yuan et al., 2018). Thus, SGK3 may be involved in maintaining the integrity of both the charge and the molecular barrier of podocytes. Thus, these data may provide new insights into how SGK3 regulates podocyte function. We previously reported that the inhibition of SGK3 resulted in decreased protein levels of podocin and CD2AP, two other slit diaphragm proteins of podocytes; however, whether SGK3 is involved in the regulation of ubiquitin-mediated degradation of podocin and CD2AP is unclear.

With respect to the detailed mechanisms of SGK3-regulated nephrin degradation, we noted that both Nedd4-2 and GSK3β were involved in SGK3-triggered nephrin levels. Nedd4-2 is an E3 ubiquitin ligase, which mainly mediates the ubiquitination-mediated degradation of various proteins through the WW domains that recognize and bind to the PPxY sequence of substrate proteins (Abriel and Staub, 2005; Rotin and Kumar, 2009). Although there is no PPxY sequence in nephrin, Nedd4-2 could interact with nephrin directly and regulate nephrin ubiquitination degradation, suggesting that Nedd4-2 regulates nephrin degradation through other motifs. This result is consistent with those reported by Sorkina et al. (2006) and Yip et al. (2016). Using the Ubi-Browser web tool, we found that Nedd4-2 can potentially interact with the Ig-like domains of nephrin, reinforcing the notion that Nedd4-2 could regulate the ubiquitination of nephrin, independent of the PPxY sequence. GSK3β, another target protein of SGK3, was reported to be involved in β-catenin and myc degradation by the proteasome pathway (Cohen and Goedert, 2004); however, we found that GSK3β did not mediate the protein degradation of nephrin through the proteasome pathway. Consistent with a previous report that the activation of GSK3 could inhibit nephrin levels *in vivo* (Boini et al., 2009), the inhibition of GSK3β through activation of SGK3 partially reversed the ADR-induced decrease in nephrin protein levels *in vitro*. Although multiple studies have shown that GSK3β regulates the nuclear transcription of nephrin through the transcriptional regulator *Snail* (Matsui et al., 2007; Wan et al., 2016; Wang et al., 2017), Cohen and Goedert (2004) reported that GSK3β could phosphorylate substrates at the serine or threonine residues, and that there are several GSK3β recognition sites in the amino acid sequence of nephrin. The precise mechanism by which GSK3β regulates nephrin requires further study.

In conclusion, SGK3 was found to mediate the ubiquitin-proteasome degradation of nephrin involved in ADR-induced podocyte injury. We further revealed that SGK3 regulated nephrin protein levels by affecting the activity of downstream target proteins, Nedd4-2 and GSK3β.

## Data Availability Statement

The original contributions presented in the study are included in the article/supplementary material, further inquiries can be directed to the corresponding author.

## Ethics Statement

The animal study was reviewed and approved by Animal Care and Use Committee of Tongji Medical College, Huazhong University of Science and Technology (approval numbers: S2433).

## Author Contributions

L-JY designed the research. Q-QD, Z-FL, HZ, H-PS, Y-CT, and Q-QL performed the experiments and analyzed the data. Q-QD, Z-FL, and L-JY wrote the manuscript. All authors approved the final version of this manuscript.

## Conflict of Interest

The authors declare that the research was conducted in the absence of any commercial or financial relationships that could be construed as a potential conflict of interest.

## Publisher’s Note

All claims expressed in this article are solely those of the authors and do not necessarily represent those of their affiliated organizations, or those of the publisher, the editors and the reviewers. Any product that may be evaluated in this article, or claim that may be made by its manufacturer, is not guaranteed or endorsed by the publisher.

## Footnotes

1
http://ubibrowser.ncpsb.org.cn/

2
http://ubibrowser.ncpsb.org/
